# Comparative effects of SGLT2 inhibitors and incretin-based therapies on dementia risk in type 2 diabetes: a systematic review and meta-analysis

**DOI:** 10.3389/fendo.2025.1695075

**Published:** 2025-10-29

**Authors:** Kirim Song, Jiwon Choi, Dayeon Jeong, Dongyun Shin, Young-Mi Ah, Ki Young Lee, Kyung Hee Choi

**Affiliations:** ^1^ College of Pharmacy, Gachon University, Incheon, Republic of Korea; ^2^ Department of Pharmacy, Chonnam National University Hwasun Hospital, Hwasun, Republic of Korea; ^3^ Department of Pharmacy, Samsung Medical Center, Seoul, Republic of Korea; ^4^ College of Pharmacy, Yeungnam University, Gyeongsan, Republic of Korea; ^5^ Department of Internal Medicine, Gachon University Gil Medical Center, Incheon, Republic of Korea; ^6^ Department of Internal Medicine, Gachon University School of Medicine, Incheon, Republic of Korea; ^7^ Department of Pharmacy, Gachon University Gil Medical Center, Incheon, Republic of Korea

**Keywords:** sodium-glucose transporter 2 inhibitors, incretins, type 2 diabetes, dementia, cognitive impairment

## Abstract

**Background:**

Antidiabetic drugs lower blood glucose levels and may also have neuroprotective and vascular protection effects. In particular, sodium–glucose cotransporter 2 inhibitors (SGLT2is) and incretin mimetics have demonstrated dementia-reducing effects. We evaluated whether SGLT2is reduce dementia risk compared with incretin mimetics in patients with type 2 diabetes (T2D).

**Methods:**

Systematic review and meta-analysis were performed by searching the PubMed, Embase, and Cochrane Library databases through February 2025. Both randomized trials and cohort studies were identified and qualitatively assessed, but only cohort studies were included in the quantitative meta-analysis. We also compared the effects of SGLT2is with those of dipeptidyl peptidase-4 inhibitors (DPP-4i) or glucagon-like peptide-1 receptor agonists (GLP-1RA) on dementia incidence.

**Results:**

Nine studies were identified for analysis. Compared with incretin mimetics, SGLT2is significantly reduced the overall dementia risk [hazard ratio (HR) 0.82, 95% CI: 0.73-0.91], and SGLT2is had stronger effects than DPP-4i (HR 0.67, 95% CI: 0.59-0.77) and GLP-1RA (HR 0.93, 95% CI: 0.86-1.00). SGLT2i also reduced the risks of vascular dementia and Alzheimer’s disease (HR 0.49, 95% CI: 0.35–0.70 vs. HR 0.68, 95% CI: 0.52–0.88, respectively). The results of subgroup analyses revealed increased benefits for patients aged older than 65 years. Empagliflozin was the most consistently protective among the SGLT2i agents.

**Conclusion:**

SGLT2is may provide neuroprotective benefits beyond glycemic control in patients with T2D, particularly in older populations at higher risk of cognitive decline. These findings support consideration of SGLT2is as a preferred therapeutic option for patients with T2D at increased risk of dementia, although randomized controlled trials would further strengthen this evidence base.

**Systematic Review Registration:**

https://www.crd.york.ac.uk/prospero/display_record.php?ID=CRD42024567890 identifier PROSPERO (CRD420251037959).

## 1 Introduction

Type 2 diabetes (T2D) is a chronic metabolic disorder; its prevalence is rapidly increasing worldwide, particularly given the aging of the population (1). Characterized by insulin resistance and chronic hyperglycemia, T2D can lead to a wide range of complications such as cardiovascular disease, kidney dysfunction, and neuropathy (2). Beyond these well-known complications, T2D substantially increases the risk of neurodegenerative disorders, such as dementia (3), which occurs approximately 1.5 times more frequently in patients with diabetes than in those without diabetes (4, 5). Globally, more than 500 million people are currently living with T2D, and the number is projected to rise to 643 million by 2030, with dementia affecting over 55 million people worldwide. This imposes significant healthcare and economic burdens on aging societies (6, 7).

Sodium-glucose cotransporter 2 inhibitors (SGLT2is) are antidiabetic agents that provide cardiovascular and renal protective effects beyond glycemic control (8, 9), and emerging evidence suggests potential neuroprotective benefits, reducing the risk of dementia and mortality in older adults (10, 11). Similarly, incretin-based therapies, including dipeptidyl peptidase-4 inhibitors (DPP-4i) and glucagon-like peptide-1 receptor agonists (GLP-1RA), have also shown potential cognitive benefits in some studies (12, 13).

Several retrospective cohort studies have investigated the association between SGLT2i use and dementia incidence, with most studies demonstrating a lower risk of dementia in SGLT2i users than in non-SGLT2i users, although some studies failed to achieve statistical significance (11, 14–22). A recent meta-analysis of these observational studies further supported the association between SGLT2i use and lower incidence of dementia compared with other antidiabetic medications (23, 24). However, these comparisons with non-SGLT2i users limit direct drug-to-drug comparative interpretation and the ability to draw robust conclusions about drug-specific neuroprotective effects (24, 25). To address this limitation, incretin-based therapies were selected as suitable references because they are known to possess potential neuroprotective properties (26), making them more clinically relevant for comparison than placebo or mixed control groups.

Our aim in this study was to conduct a systematic review and meta-analysis of the literature to evaluate the effects of SGLT2is on cognitive decline and preventing dementia in patients with T2D, using incretin mimetics for comparison. This comparative approach not only advances our scientific understanding of drug-specific neuroprotective effects but also offers practical insights that may guide therapeutic decision-making and optimization of treatment strategies in routine clinical practice.

## 2 Methods

### 2.1 Literature search

This study was conducted in accordance with the Preferred Reporting Items for Systematic Reviews and Meta-Analyses (PRISMA) 2020 guidelines (27) for systematic reviews and meta-analyses. The PRISMA checklist is provided in **Supplementary Table S1**, and the study protocol was registered in the PROSPERO database (Registration No: CRD420251037959). Eligibility criteria were defined according to the PICO (Population, Intervention, Comparator, Outcome) framework. The target population comprised adults (≥18 years) with T2D. The intervention of interest was treatment with SGLT2is, and the comparators were incretin-based therapies, including DPP-4i or GLP-1RAs; notably, intervention and comparator groups were interchangeable in this analysis. Primary outcomes included the incidence of all-cause dementia, while secondary outcomes comprised Alzheimer’s disease, vascular dementia, and all-cause mortality. The literature was searched using three databases, PubMed, Embase, and the Cochrane Library, to identify all relevant publications up to February 2025. Based on the PICO framework, the search terms included “sodium glucose transporter 2 inhibitors,” “glucagon-like peptide-1 receptor agonists,” “ dipeptidyl-peptidase IV inhibitors,” “dementia,” “cognitive impairment,” and “Alzheimer’s disease”. Medical Subject Heading (MeSH) terms and free text were used in parallel, and a detailed search strategy was developed to incorporate various synonyms and trade names related to antidiabetic medication and cognitive disorders, with the complete search strings for each database provided in **Supplementary Table S2**.

### 2.2 Literature selection

The inclusion criteria were as follows: First, the study population consisted of individuals aged 18 years or older who were diagnosed with T2D. Second, the intervention group included patients treated with SGLT2is, and the comparison group comprised patients receiving incretin mimetics, specifically GLP-1RA or DPP-4i. Eligible studies reported either the incidence of all-cause dementia or changes in cognitive function scores as their primary outcomes. The study designs included were randomized controlled trial (RCT) and prospective or retrospective cohort study.

The following studies were excluded: those with only the abstract available and those lacking full-text access. In addition, studies that did not involve human subjects, reported outcomes related to cognitive function or dementia, lacked sufficient data for calculating effect size, or were published in languages other than English, were also excluded.

### 2.3 Study selection, data extraction, and quality assessment

The studies were selected, data were extracted, and quality was assessed independently by two researchers (JC and KS). A third researcher (KC) mediated the final decision in cases of disagreement. The data were extracted from all studies meeting the inclusion criteria using a standardized form to obtain information on study characteristics, population demographics, cognitive assessment tools, types of interventions, baseline scores, types and dosages of medications used, comparison group settings, covariate adjustment, statistical analysis methods, and study results. Studies derived from the same national database were screened for inclusion in the analysis based on the study period or number of study participants to minimize the possibility of participant duplication among the selected studies. The risk of bias in RCTs was assessed using RoB 2.0 (28), whereas the ROBINS-I tool (29) was used for nonrandomized studies.

### 2.4 Outcomes

The primary outcome was the incidence of dementia. In addition, changes in cognitive function scores were considered in studies reporting this outcome. However, due to the limited number of such studies, these results were described qualitatively. The secondary outcomes included the incidence of dementia subtypes and all-cause mortality for the overall incretin mimetic group and individually for DPP-4i and GLP-1RA. Additionally, subgroups were analyzed to compare SGLT2is with DPP-4i and GLP-1RA, stratified by patient age, geographic region, follow-up treatment duration, and individual SGLT2i agents. Studies with longer durations were preferentially included in cases where duplication was suspected considering the possibility of overlapping participants among studies based on the same national databases (National Health Insurance Service, TriNex).

### 2.5 Statistical analysis

The effect sizes for the incidence of dementia were synthesized using HRs, and a meta-analysis was conducted using a random-effects model based on the inverse-variance method. The heterogeneity among studies was assessed using Cochran’s Q statistic and the I² value, with I² values exceeding 50% indicating significant heterogeneity. Sensitivity was analyzed using a leave-one-out approach by sequentially excluding individual studies, alternative analyses to account for duplicate data, and exclusion of studies with a high risk of bias. Publication bias was assessed using funnel plots, focusing on the primary outcome of dementia incidence. All statistical analyses were conducted using RevMan 5.4 (Review Manager version 5.4; Cochrane Collaboration).

## 3 Results

### 3.1 Literature selection

**Figure 1:**
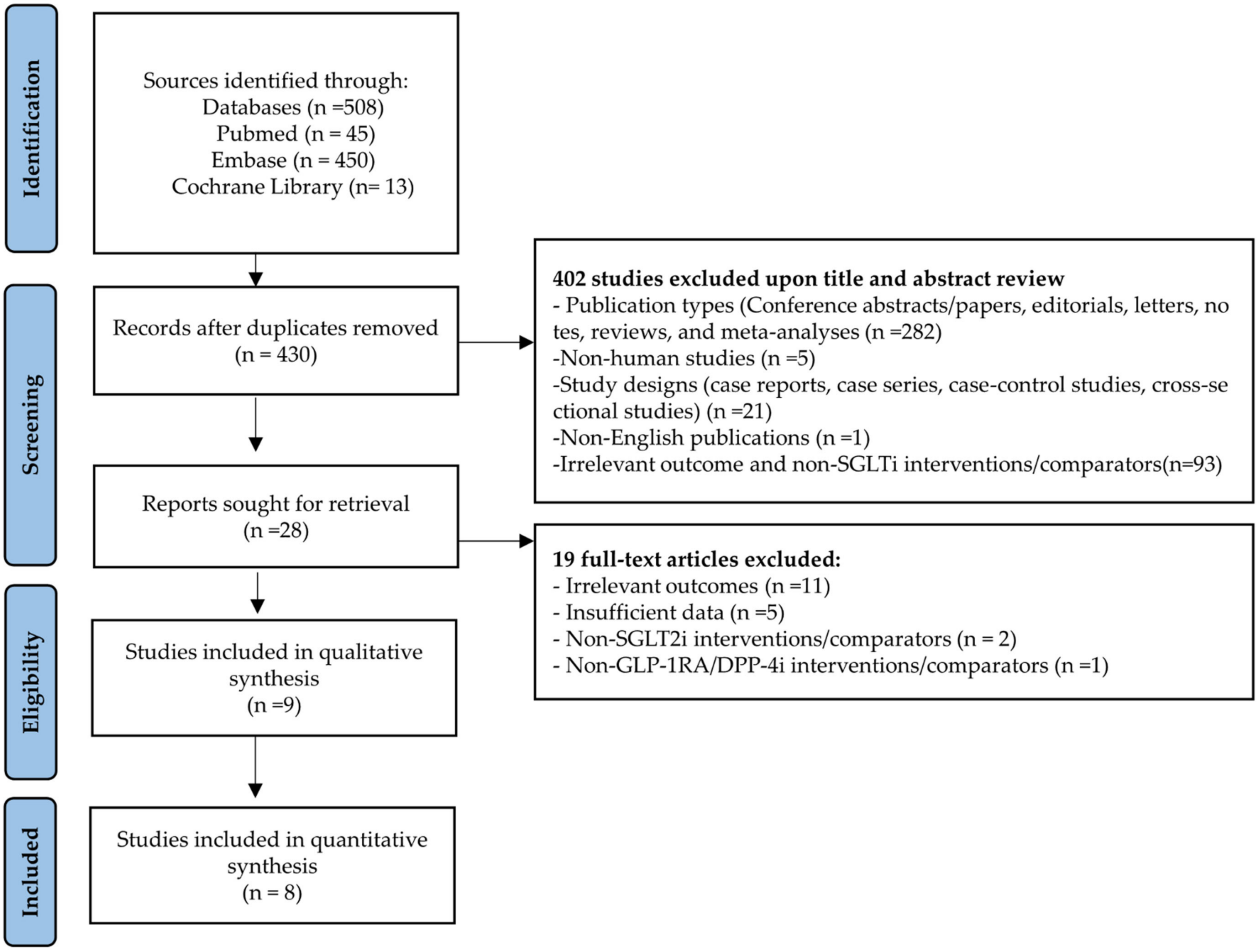
PRISMA flow diagram for study selection process.

We identified 508 publications from the initial literature search. A total of 78 duplicates and 421 studies did not meet the eligibility criteria; nine studies were thus included in this analysis (**Figure 1**). Details of the 19 excluded full-text articles are provided in **Supplementary Table S3**. Of these, eight studies were included in the quantitative synthesis. Perna et al. assessed cognitive function using neuropsychological tests, including the Verbal Fluency Test, Babcock Story Recall Test, and Attentive Matrices Test [19].

### 3.2 Characteristics of included studies

The included studies primarily evaluated dementia risk in patients with T2D by comparing SGLT2is with incretin mimetics, including DPP-4i and GLP-1RA. The included studies comprised one RCT, six population-based retrospective cohort studies, and two cohort studies emulating a target trial. The characteristics of these studies are summarized in **Table 1**; **Supplementary Table S4**. Most study populations consisted of adults aged 50 years and older, with a predominance of older adults aged over the age of 65 years in most cohorts. The proportion of female participants ranged from approximately 40% to 60%. The number of study participants varied substantially, ranging from fewer than 50 to over 390,000 individuals.

**Table 1:** Main characteristics of the included studies.

Authors (year)	Data resources	Overall study periods	Nation	Inclusion criteria	Medication, sample size, n		Age, mean ± SD, year		Women, n(%)		Outcomes
					Intervention (n)	Comparison (n)	Intervention	Comparison	Intervention	Comparison	
Randomized controlled trials											
Perna et al. (2018) (22)	EHR	2016-2017	Italy	• Age: >65 years • No prior diagnosis of dementia or MCI • Absence of acute or chronic neurological disease, mental disorder, sensorial impairment, alcoholism	SGLT2i (21) : CANA, DAPA, EMPA	Incretins (23) - *GLP-1RA:* LIRA - *DPP-4i:* VILD, SITA, LINA	77.36 ± 7.98	77.00 ± 8.73	8 (38.1 %)	8 (44.4 %)	Cognitive performance • Verbal fluency test • Babcock story recall test • Attentive matrices test
Population- based retrospective cohort study											
Abdullah et al. (2025) (15)	CPRD Aurum -database	2013-2022	UK	• Age: ≥40 years • No prior use of either drug before index date • No history of dementia, MCI, anti-dementia medications • Excluded with ESRD or dialysis	SGLT2i (34,797) : CANA,DAPA,EMPA,ERTU	DPP-4i (82,939) : NA	56.83 ± 8.96	56.91 ± 9.06	13,681 (39.3%)	33,226 (40.1%)	• New- onset of dementia • Incident MCI, AD, VD
De Giorgi et al. (2024) (16)	TriNet X	2017-2021	USA	• Age: ≥18 years • No prior use of either drug before index date	GLP-1 RA (22,584) : SEMA	SGLT2i (22,584) : EMPA	57.6 ± 12.3	57.6 ± 12.4	11,067 (49.0%)	11,012 (48.8%)	• Incident dementia based on ICD-10 code • All-cause mortality
Hong et al. (2024) (18)	NHIS-NHID	2014-2020	South Korea	• Age: ≥40 years • Excluded with ESRD or dialysis	SGLT2i (42,873) : DAPA,EMPA,ERTU,IPRA	DPP-4i (384,757) : ALOG, ANAG, EVOG, GEMI, LINA, SAXA, SITA, TENE, VILD	59.8 ± 10.5	59.8 ± 10.6	25,145 (58.6%)	225,373 (58.6%)	• New- onset of dementia
Mui et al. (2021) (19)	Clinical Data Analysis and Reporting System	2015-2019	Hong Kong	• No prior diagnosis of all-cause dementia, parkinsonism. • Excluded <1 month exposure	SGLT2i (13,283) : NA	DPP-4i (26,545) : NA	61.18 ± 3.65	62.08 ± 3.96	5,089 (38.3%)	10,831 (40.8%)	• New- onset of dementia including AD, VD • All-cause mortality, cardiovascular, and cerebrovascular mortality
Pai et al. (2024) (11)	TriNet X	2012-2022	USA	• Age: ≥50 years • No history of dementia and use of related medication	SGLT2i+MET (193,948) : NA	GLP-1RA+MET (193,948) : NA	64.02 ± 9.59	63.79 ± 10.51	86,389 (44.5%)	86,621 (44.7%)	• New- onset of dementia
					SGLT2i+MET (165,566) : NA	DPP-4i+MET (165,566) : NA	63.91 ± 9.55	63.86 ± 9.28	72,187 (43.6%)	71,341 (43.1%)	
Wu et al. (2023) (21)	Ontario Diabetes Database, ODB, OHIP	2016-2021	Canada	• Age: ≥66 years • No history of dementia	SGLT2i (36,513) : CANA, DANA, EMPA	DPP-4i (36,545) : LINA, SAXA, SITA	72.4 ± 5.38	72.41 ± 3.89	14,164 (38.8%)	14,237 (39.0%)	• Incident dementia of AD and related dementias
Population based cohort study emulating a target trial											
Hong et al. (2024) (17)	NHIS-NHID	2010-2022	South Korea	• Age: ≥60 years • Used both metformin and no use of either drug in past year • ≥2 years of potential follow-up • No history of dementia • Excluded with ESRD or dialysis	SGLT2i (2,076) : DAPA, EMPA	GLP-1RA (1,038) : DULA	66.8± 5.5	66.8± 5.5	1,013 (48.8%)	512 (49.3%)	• New- onset of dementia including AD, VD
Shin et al. (2024) (20)	NHIS-NHID	2013-2021	South Korea	• Age: 40–69 years • No history of dementia and use of related medication • No prior use of GLP-1RA or TZD	SGLT2i (110,885) : DAPA, EMPA, ERTU, IPRA	DPP-4i (110,885) : ALOG, ANAG, EVOG, GEMI, LINA, SAXA, SITA, TENE, VILD	61.9± 4.4	61.9± 4.5	49,090 (44.3%)	49,142 (44.3%)	• New- onset of dementia including AD, VD

SGLT2i; CANA, canagliflozin; DAPA, dapagliflozin; EMPA, empagliflozin; ERTU, ertugliflozin; IPRA, ipragliflozin.; DPP-4i, ALOG, alogliptin; ANAG, anagliptin; GEMI, gemigliptin; LINA, linagliptin; SAXA, saxagliptin; SITA, sitagliptin; TENE, tenegliptin; VILD, vildagliptin; GLP-1RA, DULA, dulaglutide; LIRA, liraglutide; SEMA, semaglutide; EHR, Electronic Health Records; ODB, Ontario Drug Benefit; OHIP, Ontario Health Insurance Plan; CPRD, The Clinical Practice Research Datalink; NHIS-NHID, National Health Insurance Service-National Health Insurance Database; MCI, mild cognitive impairment; AD, Alzheimer’s disease; VD, vascular dementia, ICD, International Classification of Diseases; NA, not available; TZD, thiazolidinediones; ESRD, end-stage renal disease; MET, metformin.

Five studies compared SGLT2is with DPP-4 inhibitors only, two with GLP-1 receptor agonists only, and one (11) with both. One RCT (22) compared SGLT2is with incretin-based therapies as a combined comparator group. The mean follow-up period among all studies ranged from two to five years, suggesting the need for longer observational studies for evaluating the long-term dementia-prevention effects of SGLT2is.

### 3.3 New-onset dementia

**Figure 2:**
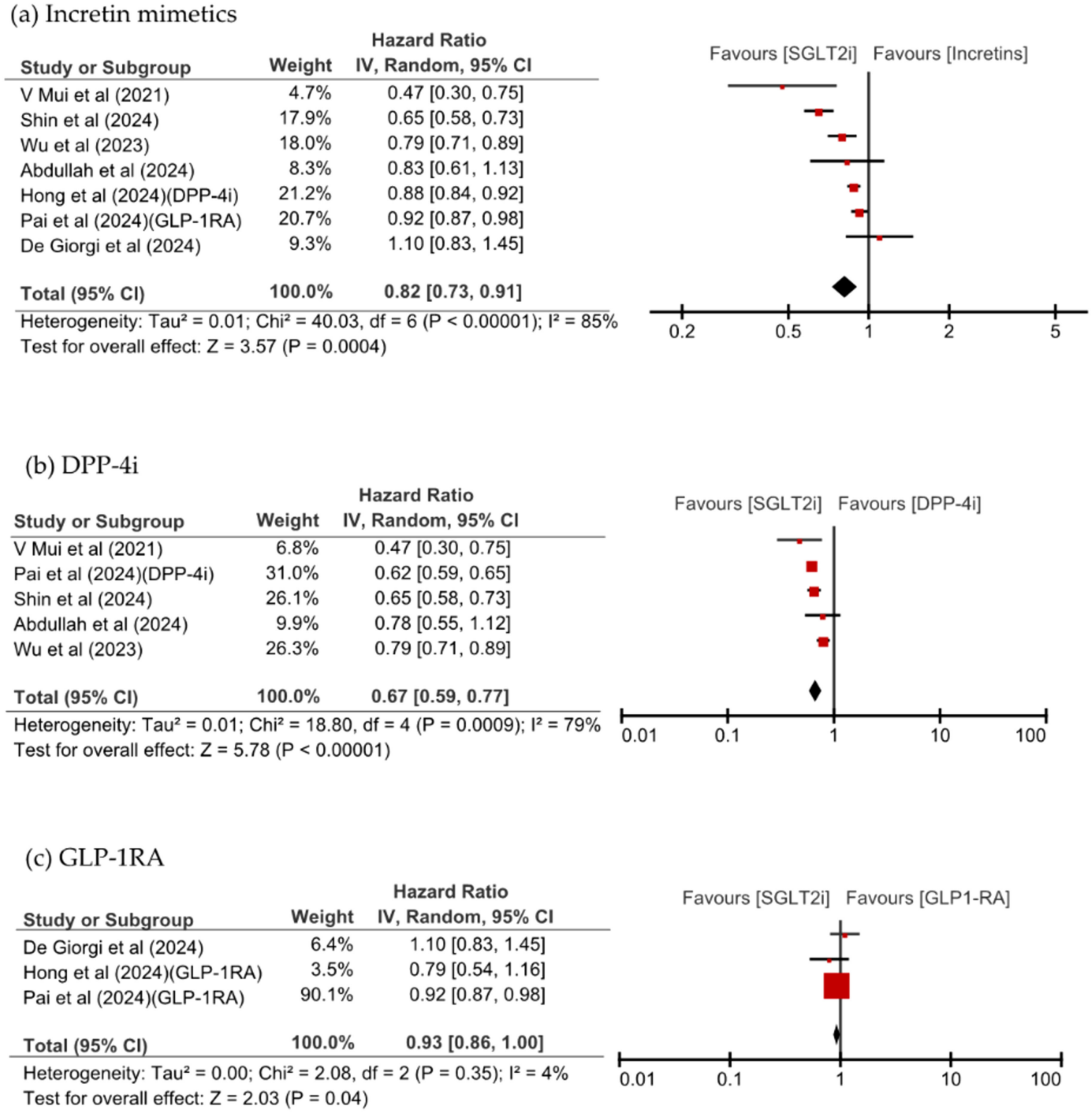
Forest plot of new-onset dementia with SGLT2is compared with **(a)** incretin mimetics, **(b)** DPP-4i, and **(c)** GLP-1RA.

To avoid overlap, only the study by Shin et al., which used a longer study period, was included among those using the same national database (excluding the study by Hong et al.), resulting in a total of seven studies included in the comparison between SGLT2is and incretin mimetics. SGLT2i use was associated with a significantly reduced overall risk of dementia (hazard ratio [HR] 0.82, 95% CI: 0.73–0.91, p = 0.0004, I^2^ = 85%). Upon separately examining each class of incretin mimetics, SGLT2is were found to be associated with a significantly lower risk of dementia than DPP-4is were (HR 0.67, 95% CI: 0.59–0.77, p <0.00001, I^2^ = 79%). Additionally, SGLT2is showed a trend toward a lower risk of dementia compared with GLP-1RA did (HR 0.93, 95% CI: 0.86–1.00, p = 0.04, I^2^ = 4%). As shown in **Figure 2**, Most studies consistently showed directional effects associating SGLT2is with a reduced risk of dementia.

### 3.4 Vascular dementia, Alzheimer’s disease, and all-cause mortality

**Figure 3:**
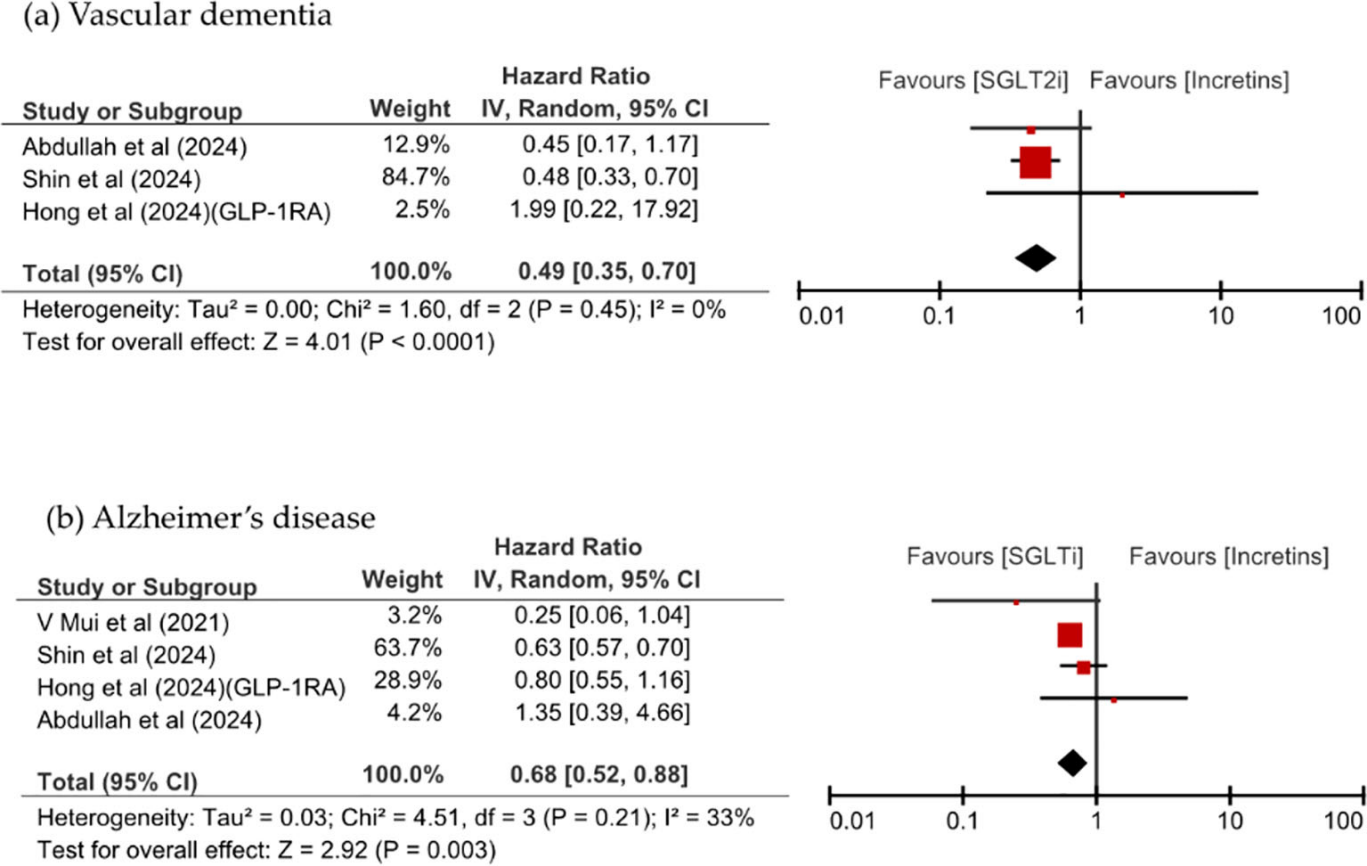
Comparison of dementia incidence between SGLT2is and incretin mimetics, stratified by dementia subtype: **(a)** vascular dementia, **(b)** Alzheimer’s disease.

**Figure 4:**
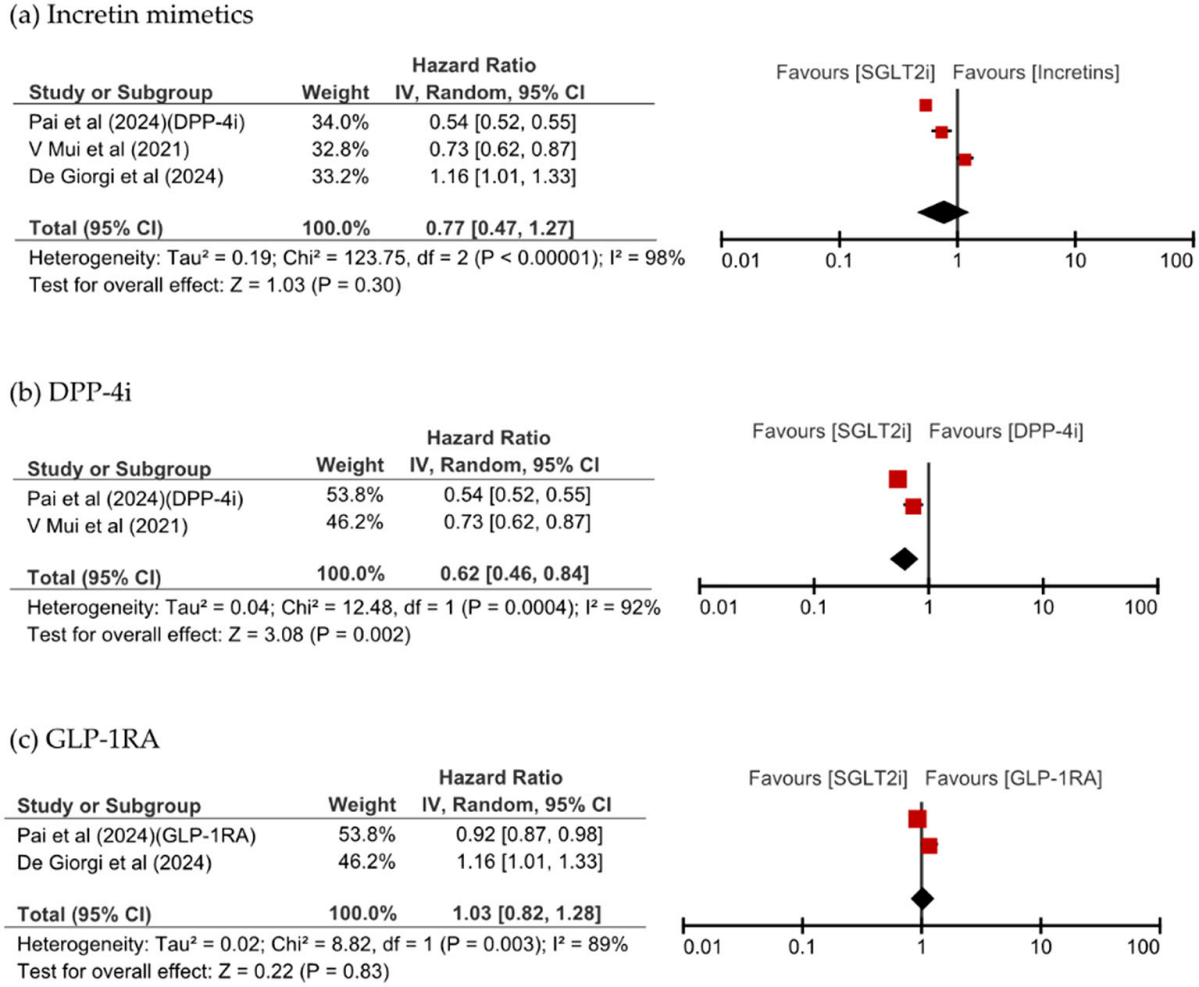
Forest plot of all-cause mortality with SGLT2is compared with **(a)** incretin mimetics, **(b)** DPP-4i, and **(c)** GLP-1RA.

**Figure 3** shows that SGLT2i use was associated with a significant reduction of VD and AD risks, in the analysis of dementia risk by specific dementia types comparing SGLT2is and incretin mimetics (HR 0.49, 95% CI: 0.35–0.70, p <0.0001, I^2^ = 0% vs. HR 0.68, 95% CI: 0.52–0.88, p = 0.003, I^2^ = 33%). SGLT2is use numerically lower all-cause mortality than incretins use; however, this difference was not significant (HR 0.77, 95% CI: 0.47–1.27, p = 0.30, I^2^ = 98%). SGLT2i use significantly reduced the risk of VD compared with DPP-4i use (HR 0.48, 95% CI: 0.34–0.68, p <0.0001, I^2^ = 0%) and significantly reduced the risk of all-cause mortality (HR 0.62, 95% CI: 0.46–0.84, p = 0.002, I^2^ = 92%). All-cause mortality was comparable between patients taking SGLT2is and those taking GLP-1RA, as shown in **Figure 4**.

### 3.5 Subgroup analysis

#### 3.5.1 Patient and study characteristics

**Figure 5:**
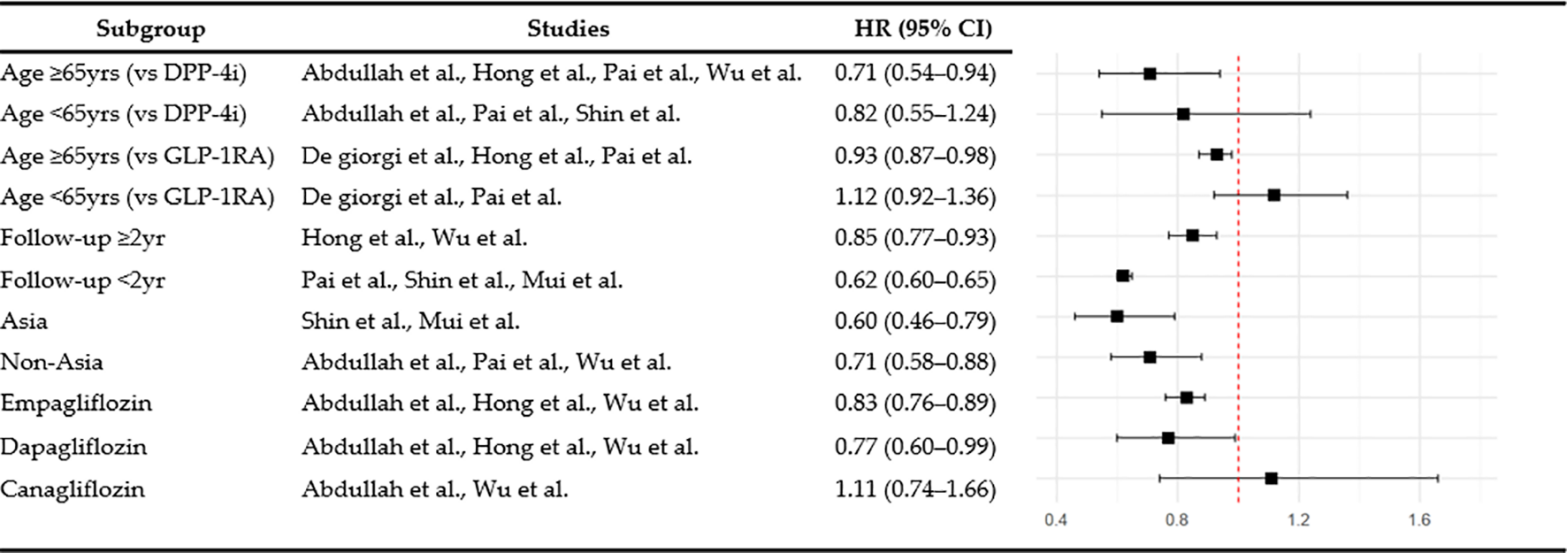
Subgroup analysis of dementia risk.

Subgroup analyses were performed to examine dementia risk according to age, follow-up duration, and study region. Among individuals aged 65 and over, SGLT2is use was associated with a significantly lower risk of dementia than GLP-1RA use (HR 0.93, 95% CI: 0.87–0.98, p = 0.008, I^2^ = 0%), whereas no significant difference was observed in the under-65 age group (HR 1.12, 95% CI: 0.92–1.36, p = 0.26, I^2^ = 0%). When stratified by follow-up duration, the dementia risk associated with SGLT2is compared with DPP-4i was 0.62 (95% CI: 0.60–0.65, p < 0.00001, I^2^ = 0%) for less than two years and 0.85 (95% CI: 0.77–0.93, p = 0.0006, I^2^ = 62%) for two or more years. In the regional subgroup analysis, SGLT2is use was associated with a significantly lower risk of dementia in Asian populations (HR 0.60, 95% CI: 0.46–0.79, p = 0.0002, I^2^ = 42%), and also significant among non-Asian populations (HR 0.71, 95% CI: 0.58-0.88, p = 0.002, I^2^ = 88%). The overall findings from these subgroup analyses are summarized in **Figure 5**.

#### 3.5.2 Individual SGLT2i agents

Empagliflozin significantly reduced the risk of dementia compared with DPP-4i (HR 0.83, 95% CI: 0.76–0.89, p <0.00001, I^2^ = 6%) based on the results of a subgroup analysis by individual agents within the SGLT2i class. Dapagliflozin (HR 0.77, 95% CI: 0.60–0.99, p = 0.05, I^2^ = 73%) showed borderline significance for the risk of dementia. In contrast, the effect of canagliflozin did not reach statistical significance (HR 1.11, 95% CI: 0.74–1.66, p = 0.61, I^2^ = 56%).

### 3.6 Quality and sensitivity analyses

The results of the quality assessment of the final selected studies are presented in **Supplementary Tables S5**, **S6**. In the included RCT, the study by Perna et al. (22) was rated as having “some concerns,” primarily due to issues related to the randomization process and outcome measurement. One non-randomized study by Abdullah et al. (15) was assessed as having a critical risk of bias, mainly due to the presence of confounding effects. All other non-RCTs were evaluated as having a moderate risk of bias. Furthermore, no evidence of publication bias was detected based on visual inspection of the funnel plot (**Supplementary Figure S4**). Although a meta-regression was considered to explore potential sources of heterogeneity, it could not be performed because of the limited number of studies within each subgroup (30, 31). We reanalyzed the data by including studies that were previously excluded because of the potential overlap in study populations to assess the robustness of the main findings, (Pai et al. and Hong et al. [GLP-1RA]). The direction and significance of these effects were consistent. The leave-one-out analysis, in which each study was sequentially removed, also yielded consistent results: SGLT2is significantly reduced the risk of dementia compared with incretin mimetics across all analyses, as shown in **Supplementary Figures S2**, **S3**. Additional sensitivity analyses using intention-to-treat and as-treated approaches further supported the robustness of the results (**Supplementary Figure S1**). Although a meta-regression was considered to explore potential sources of heterogeneity, it could not be performed because of the limited number of studies within each subgroup.

Perna et al. administered SGLT2is (n=21) and incretin mimetics (n=18) for 12 months to older adults with T2D with a mean age of 77 years; no significant changes were observed in various cognitive function assessments, including the Verbal Fluency Test, Attentive Matrices Test, and Babcock Story Recall Test in either group (p > 0.05).

## 4 Discussion

The pathophysiological mechanisms underlying the association between T2D and dementia involve multiple interconnected pathways. Chronic hyperglycemia promotes the accumulation of advanced glycation end-products, which trigger β-amyloid deposition and neurofibrillary tangle formation, ultimately leading to neuronal damage (32). Furthermore, insulin resistance in neural cells disrupts normal insulin signaling, and impairs the suppression of β-amyloid production and tau protein hyperphosphorylation, promoting cognitive decline (33). Moreover, diabetes-associated microvascular dysfunction, oxidative stress, and low-grade systemic inflammation may exacerbate blood–brain barrier (BBB) disruption and neuroinflammatory responses, further contributing to the progression of dementia (34, 35).

We found that compared with incretin mimetics, SGLT2is significantly reduced the risk of overall incidence of dementia, VD, and AD. These findings suggest neuroprotective effects beyond glucose control, supported by preclinical and clinical studies (10, 11, 23, 36). Preclinical studies demonstrate that SGLT2is prevent structural damage and inhibit ultrastructural changes associated with cognitive decline in the neurovascular units of a diabetic mouse model (37). Reductions in β-amyloid deposition and tau protein phosphorylation were observed in the brain tissues of mice with both AD and T2D (38). Additionally, SGLT2is may modulate the AMPK/mTOR pathway and promote autophagy (39). Beyond these direct neuroprotective effects, SGLT2is exert anti-inflammatory effects that mitigate systemic and neuroinflammatory processes that contribute to cognitive decline (40, 41). Subsequently, multiple clinical studies have reported the effects of SGLT2is on dementia (9, 42, 43).

Both DPP-4i and GLP-1RA modulate the incretin hormone GLP-1 but differ in their neuroprotective mechanisms. GLP-1RA directly acts on GLP-1 receptors and can cross the BBB enabling direct central nervous system effects including anti-inflammatory, antioxidant, and β-amyloid clearance effects (44). In contrast, DPP-4i indirectly increases GLP-1 and glucose-dependent insulinotropic polypeptide levels without direct brain penetration (45). These mechanistic differences are reflected in clinical outcomes. GLP-1RAs have demonstrated consistent and robust cognitive benefits (46–48), with up to 53% in RCT and 27% in case–control studies, compared with placebo (49), while DPP-4i exhibited mixed results (50–53). Our finding that SGLT2is was more effective than DPP-4i but comparable to GLP-1RA (HR 0.93, 95% CI: 0.86–1.00, p = 0.04, I^2^ = 4%) align with these mechanistic differences. The pronounced neuroprotective effect of GLP-1RA may have attenuated the observed relative effect size of SGLT2is in direct comparisons, indirectly suggesting that SGLT2is are likely to exert neuroprotective effects of comparable clinical relevance.

Perna et al. observed no deterioration in cognitive function following at least one year of treatment with either SGLT2is or incretin mimetics, as assessed using standardized neuropsychological measures. These findings suggest that at least 12 months of treatment with these agents does not detrimentally affect cognitive function. Although the study was conducted in a population with normal baseline cognitive function, these findings suggest that these agents maintain cognitive stability.

In subgroup analysis, SGLT2i use significantly reduced the risk of dementia in patients aged 65 and older, whereas no significant effect was observed in those under 65 years, when compared with DPP-4i use. The age-related benefit may reflect physiological differences that may enhance the effects of the drugs in older adults, who are at high risk of dementia. Age-related deterioration in insulin sensitivity, elevated oxidative stress, and compromised mitochondrial function collectively increase susceptibility to cognitive impairment in older populations (54–56), potentially making older adults more responsive to the protective mechanism of SGLT2is. This finding aligns with those of prior studies, suggesting that the cardiovascular and metabolic benefits of SGLT2is are more evident in older patients (57–59). SGLT2is showed consistent protective effects against dementia regardless of the follow-up period. Notably, a significant reduction in dementia risk was observed even in the short-term follow-up group of less than 2 years, suggesting that SGLT2is may exert cognitive protective effects within a relatively short period. This early benefit may be attributable to the rapid improvements in inflammatory responses, vascular function, and oxidative stress that provide immediate neuroprotective effects. Longer follow-up studies have shown cumulative protective effects, underscoring the importance of both early and sustained SGLT2i use. However, some cases of dementia diagnosed within a short period after drug administration cannot exclude the possibility of reverse causality; thus, additional research is required considering the slow progression of dementia and importance of lag-time settings. The results of subgroup analysis by region showed that SGLT2is had significant dementia-prevention effects in Asian and non-Asian populations, suggesting that the cognitive benefits of SGLT2is may extend across diverse ethnic and regional groups.

Distinct trends in the reduction of dementia risk emerged when individual SGLT2i agents were compared with DPP-4i. Empagliflozin had a consistent protective effect with low heterogeneity, whereas dapagliflozin had a protective effect with borderline significance and canagliflozin did not achieve statistical significance. These findings suggest that empagliflozin may be relatively more effective in preserving cognitive function. Although canagliflozin’s lack of significance may partly reflect its substantially smaller sample size across studies, differences in pharmacological properties among SGLT2i agents may also contribute to the varying neuroprotective effects. Empagliflozin’s superior cognitive protective effects may be related to its high SGLT2 selectivity and BBB permeability, which enables effective inhibition of microglial overactivation and subsequent protection of neurons and glial cells (60). In support of this, empagliflozin reduced the levels of both the neuronal damage marker neurofilament light chain and glial damage marker S100BB. In contrast, canagliflozin’s lower SGLT2 selectivity and dual inhibition of SGLT1 and SGLT2 may have limited its central nervous system penetration. While canagliflozin reduced neurofilament light chain levels, its inconsistent effects on S100BB suggest limited glial protection (61). This distinction has clinical relevance because glial cell hyperactivation induces central nervous system inflammation in major neurodegenerative disorders, including AD and Parkinson’s disease (62). These findings suggest that the neuroprotective potential of individual SGLT2i agents depends on their BBB permeability as well as their capacity for neuroglial protection, factors that should be considered in clinical decision-making.

This study has a few limitations. First, to avoid data duplication, only one study was selected from those using the same database when researcher overlap was possible. Although this approach helped maintain data independence, it may have reduced the total number of studies included, potentially limiting the representativeness of our findings. Second, because this meta-analysis included only cohort studies, causal relationships could not be definitively established. Although most of the included studies were of high quality and adjusted for major confounders such as age, sex, body mass index, glycemic parameters, hypertension, and other cardiometabolic factors, these adjustments were not fully consistent across studies. To address these limitations and assess the robustness of our findings, we conducted extensive subgroup and sensitivity analyses—including leave-one-out testing and reanalysis of previously excluded studies—which consistently confirmed both the direction and statistical significance of the association between SGLT2is use and reduced dementia risk. Thirdly, potential publication bias was evaluated using funnel plots. Visual inspection revealed no substantial asymmetry, suggesting a low likelihood of publication bias. However, given that fewer than ten studies were included in the meta-analysis, this finding should be interpreted with caution. Finally, although one RCT was identified in our systematic review, it was not included in the meta-analysis due to differences in outcome measures. Nevertheless, the findings from this RCT provide meaningful insights into the cognitive outcomes associated with these agents.

This study is meaningful in its comparison of SGLT2i users with those using other antidiabetic agents known for their cognitive benefits. SGLT2is were found to be noninferior to GLP-1RA, which have cognitive effects; we also identified differences in effects among SGLT2i agents. Furthermore, we observed an association between SGLT2i use and a reduced risk of dementia in older adults aged 65 years and above, with no evidence of racial disparities. These results have important clinical implications for T2D management in patients at risk of cognitive decline. First, the demonstrated neuroprotective effects support expanding treatment selection criteria beyond glycemic control to include cognitive outcomes, particularly in older adults. Second, empagliflozin’s superior performance suggests that individual agent selection may be clinically meaningful when cognitive protection is a treatment goal. Third, the early onset of protective effects observed within two years indicates that timely initiation of SGLT2is therapy may provide immediate cognitive benefits alongside long-term cumulative effects. For clinical practice, these findings suggest incorporating cognitive risk assessment into diabetes care frameworks and considering SGLT2is as preferred agents for patients with elevated dementia risk. While awaiting confirmatory RCTs, the present evidence supports SGLT2is as a rational choice for comprehensive diabetes management that addresses both metabolic and cognitive outcomes. Future prospective studies should employ rigorous comparative designs with extended observation periods and careful adjustment for baseline patient factors to establish definitive causal relationships.

## 5 Conclusions

The results of this meta-analysis suggest that compared with incretin-based therapies, the use of SGLT2is was associated with a potential reduction in overall risk of dementia, with additional benefits observed in both VD and AD. Among SGLT2is, empagliflozin appeared to show a relatively stronger association, although this finding should be interpreted with caution due to the limited number of studies and potential differences in study design. The observed cognitive effects of SGLT2is were consistent across various subgroups, including older age groups, all follow-up durations, and diverse racial groups. Given the limitations of observational studies including heterogeneity and potential bias, future RCT are warranted. Large-scale, head-to-head studies with standardized methodologies and long-term follow-up will be required, specifically accounting for patient characteristics, comorbidities, concomitant medications, and age-specific effects to confirm the cognitive protective effects of SGLT2is.

## Data Availability

The original contributions presented in the study are included in the article/**Supplementary Material**. Further inquiries can be directed to the corresponding author.

## Supplementary material

Supplementary Table 1 Checklist of preferred reporting items for systematic reviews and meta-analyses.

Supplementary Table 2 Search strategy.

Supplementary Table 3 List of excluded full-text articles with reasons for exclusion.

Supplementary Table 4 Detailed study protocol and baseline population characteristics of included studies.

Supplementary Table 5 Detailed information on the risk of bias assessment according to the Risk of Bias 2 (ROB2) criteria.

Supplementary Table 6 Detailed information regarding the risk of bias assessment according to the Risk of Bias in Non-randomized Studies-of Interventions (ROBINS-I).

Supplementary Figure 1 Subgroup analysis of dementia risk: Intention-to-Treat (ITT) vs. As-Treated (AT) approaches.

Supplementary Figure 2 Sensitivity analysis of new-onset dementia outcome.

Supplementary Figure 3 Sensitivity analysis excluding studies with high risk of bias.

Supplementary Figure 4 Funnel plot of included studies evaluating SGLT2is versus incretin-based therapies for incident dementia.
