# Supplementary material for: Comparative effects of SGLT2 inhibitors and incretin-based therapies on dementia risk in type 2 diabetes: a systematic review and meta-analysis
Source: Front Endocrinol (Lausanne). 2025 Oct 29;16:1695075. doi: 10.3389/fendo.2025.1695075 (PMC12605505; doi:10.3389/fendo.2025.1695075)
Supplement: Supplementary Table 1 — Checklist of preferred reporting items for systematic reviews and meta-analyses. [file DataSheet1.docx]

Supplementary Material

# Supplementary Tables

**Supplementary Table S1.** Checklist of preferred reporting items for systematic reviews and meta-analyses

| **Section/topic** | **#** | **Checklist item** | **Reported on page #** |
| --- | --- | --- | --- |
| **TITLE** |  |  |  |
| Title | 1 | Identify the report as a systematic review, meta-analysis, or both. | 1 |
| **ABSTRACT** |  | |  |
| Structured summary | 2 | Provide a structured summary including, as applicable: background; objectives; data sources; study eligibility criteria, participants, and interventions; study appraisal and synthesis methods; results; limitations; conclusions and implications of key findings; systematic review registration number. | 2 |
| **INTRODUCTION** |  |  |  |
| Rationale | 3 | Describe the rationale for the review in the context of what is already known. | 3 |
| Objectives | 4 | Provide an explicit statement of questions being addressed with reference to participants, interventions, comparisons, outcomes, and study design (PICOS). | 3 |
| **METHODS** | | | |
| Protocol and registration | 5 | Indicate if a review protocol exists, if and where it can be accessed (e.g., Web address), and, if available, provide registration information including registration number. | 2-3 |
| Eligibility criteria | 6 | Specify study characteristics (e.g., PICOS, length of follow-up) and report characteristics (e.g., years considered, language, publication status) used as criteria for eligibility, giving rationale. | 3 |
| Information sources | 7 | Describe all information sources (e.g., databases with dates of coverage, contact with study authors to identify additional studies) in the search and date last searched. | 3-4 |
| Search | 8 | Present full electronic search strategy for at least one database, including any limits used, such that it could be repeated. | Supplementary Table S2 |
| Study selection | 9 | State the process for selecting studies (i.e., screening, eligibility, included in systematic review, and, if applicable, included in the meta-analysis). | Figure 1. |
| Data collection process | 10 | Describe method of data extraction from reports (e.g., piloted forms, independently, in duplicate) and any processes for obtaining and confirming data from investigators. | 4 & Supplementary Table S3 |
| Data items | 11 | List and define all variables for which data were sought (e.g., PICOS, funding sources) and any assumptions and simplifications made. | 3-4 |
| Risk of bias in individual studies | 12 | Describe methods used for assessing risk of bias of individual studies (including specification of whether this was done at the study or outcome level), and how this information is to be used in any data synthesis. | 3-4 |
| Summary measures | 13 | State the principal summary measures (e.g., risk ratio, difference in means). | 3-4 |
| Synthesis of results | 14 | Describe the methods of handling data and combining results of studies, if done, including measures of consistency (e.g., I^2^) for each meta-analysis. | 4-5 |
| Risk of bias across studies | 15 | Specify any assessment of risk of bias that may affect the cumulative evidence (e.g., publication bias, selective reporting within studies). | 4-5 |
| Additional analyses | 16 | Describe methods of additional analyses (e.g., sensitivity or subgroup analyses, meta-regression), if done, indicating which were pre-specified. | 4-5 |
| **RESULTS** | | | |
| Study selection | 17 | Give numbers of studies screened, assessed for eligibility, and included in the review, with reasons for exclusions at each stage, ideally with a flow diagram. | 5 |
| Study characteristics | 18 | For each study, present characteristics for which data were extracted (e.g., study size, PICOS, follow-up period) and provide the citations. | 5 |
| Risk of bias within studies | 19 | Present data on risk of bias of each study and, if available, any outcome level assessment (see item 12). | Supplementary Table T5, T6 |
| Results of individual studies | 20 | For all outcomes considered (benefits or harms), present, for each study: (a) simple summary data for each intervention group (b) effect estimates and confidence intervals, ideally with a forest plot. | Figure 2-5 |
| Synthesis of results | 21 | Present results of each meta-analysis done, including confidence intervals and measures of consistency. | 5-6 |
| Risk of bias across studies | 22 | Present results of any assessment of risk of bias across studies (see Item 15). | Supplementary Figure S4 |
| Additional analysis | 23 | Give results of additional analyses, if done (e.g., sensitivity or subgroup analyses, meta-regression [see Item 16]). | 5-6 & Supplementary Figure S1 |
| **DISCUSSION** |  |  |  |
| Summary of evidence | 24 | Summarize the main findings including the strength of evidence for each main outcome; consider their relevance to key groups (e.g., healthcare providers, users, and policy makers). | 7-8 |
| Limitations | 25 | Discuss limitations at study and outcome level (e.g., risk of bias), and at review-level (e.g., incomplete retrieval of identified research, reporting bias). | 8-9 |
| Conclusions | 26 | Provide a general interpretation of the results in the context of other evidence, and implications for future research. | 9 |
| **FUNDING** |  |  |  |
| Funding | 27 | Describe sources of funding for the systematic review and other support (e.g., supply of data); role of funders for the systematic review. | 10 |

**Supplementary Table S2**. Search strategy

| **Pubmed** | | |
| --- | --- | --- |
| **No.** | **PICO** | **Search History** |
| #1 | Patient | "Diabetes mellitus"[MeSH Terms] OR "Type 2 diabetes mellitus" OR "T2DM" |
|  | Intervention Comparison | **Intervention** |
| #2 |  | "sodium glucose transporter 2 inhibitors"[MeSH Terms] OR "Sodium-Glucose CoTransporter 2 Inhibitor*" OR "Sodium-Glucose Co Transporter 2 Inhibitor*" OR "sglt2i*" OR "gliflozin*" OR "canagliflozin" OR "invokana" OR "Dapagliflozin" OR "farxiga" OR "forxiga" OR "empagliflozin" OR "jardiance" OR "ertugliflozin" OR "steglatro" OR "sotagliflozin" OR "inpefa" |
|  |  | **Comparison** |
| #3 |  | "Glucagon-Like Peptide-1 Receptor Agonists"[MeSH Terms] OR "glp 1 receptor agonist*" OR "incretin mimetic*" OR "glp 1 analog*" OR "Liraglutide" OR "Victoza" OR "Semaglutide" OR "Ozempic" OR "rybelsus" OR "Dulaglutide" OR "Trulicity" OR "Exenatide" OR "Byetta" OR "Bydureon" OR "Exendin 4" OR "Dipeptidyl-Peptidase IV Inhibitors"[MeSH Terms] OR "DPP4 Inhibitor*" OR "Gliptin*" OR "sitagliptin" OR "Januvia" OR "saxagliptin" OR "onglyza" OR "linagliptin" OR "trajenta" OR "alogliptin" OR "nesina' OR "anagliptin" OR "guardlet" OR "evogliptin" OR "suganon" OR "gemigliptin" OR "zemiglo" OR "tenegliptin" OR "tenelia" OR "vidagliptin" OR "galvus" |
| #4 |  | #2 AND #3 |
| #5 | Outcome | "cogniti*" OR "Alzheimer*" OR "dementia*" |
|  | Total | #1 AND #4 AND #5 |

| **Embase** | | |
| --- | --- | --- |
| **No.** | **PICO** | **Search History** |
| #1 | Patient | diabetes mellitus'/exp OR 'Type 2 diabetes mellitus' OR 'T2DM' |
|  | Intervention Comparison | **Intervention** |
| #2 |  | sodium-glucose transporter 2 inhibitors'/exp OR canagliflozin'/exp OR dapagliflozin'/exp OR empagliflozin'/exp OR ertugliflozin' /exp OR sotagliflozin' /exp |
|  |  | **Comparison** |
| #3 |  | Glucagon-Like Peptide-1 Receptor Agonists'/exp OR liraglutide' /exp OR semaglutide'/exp OR dulaglutide' /exp OR exenatide'/exp OR Dipeptidyl-Peptidase IV Inhibitors'/exp OR sitagliptin' /exp OR saxagliptin' /exp OR linagliptin' /exp OR alogliptin' /exp OR anagliptin' /exp OR evogliptin'/exp OR gemigliptin' /exp OR tenegliptin' /exp OR vidagliptin'/exp |
| #4 |  | #2 AND #3 |
| #5 | Outcome | cogniti*' OR Alzheimer*" OR "dementia' |
|  | Total | #1 AND #4 AND #5 |
| **Cochrane Library** | | |
| **No.** | **PICO** | **Search History** |
| #1 | Patient | "Diabetes mellitus"[MeSH Terms] OR "Type 2 diabetes mellitus" OR "T2DM" |
|  | Intervention Comparison | **Intervention** |
| #2 |  | "sodium glucose transporter 2 inhibitors"[MeSH Terms] OR "Sodium-Glucose CoTransporter 2 Inhibitor*" OR "Sodium-Glucose Co Transporter 2 Inhibitor*" OR "sglt2i*" OR "gliflozin*" OR "canagliflozin" OR "invokana" OR "Dapagliflozin" OR "farxiga" OR "forxiga" OR "empagliflozin" OR "jardiance" OR "ertugliflozin" OR "steglatro" OR "sotagliflozin" OR "inpefa" |
|  |  | **Comparison** |
| #3 |  | "Glucagon-Like Peptide-1 Receptor Agonists"[MeSH Terms] OR "glp 1 receptor agonist*" OR "incretin mimetic*" OR "glp 1 analog*" OR "Liraglutide" OR "Victoza" OR "Semaglutide" OR "Ozempic" OR "rybelsus" OR "Dulaglutide" OR "Trulicity" OR "Exenatide" OR "Byetta" OR "Bydureon" OR "Exendin 4" OR "Dipeptidyl-Peptidase IV Inhibitors"[MeSH Terms] OR "DPP4 Inhibitor*" OR "Gliptin*" OR "sitagliptin" OR "Januvia" OR "saxagliptin" OR "onglyza" OR "linagliptin" OR "trajenta" OR "alogliptin" OR "nesina' OR "anagliptin" OR "guardlet" OR "evogliptin" OR "suganon" OR "gemigliptin" OR "zemiglo" OR "tenegliptin" OR "tenelia" OR "vidagliptin" OR "galvus" |
| #4 |  | #2 AND #3 |
| #5 | Outcome | "cogniti*" OR "Alzheimer*" OR "dementia*" |
|  | Total | #1 AND #4 AND #5 |

**Supplementary Table S3**. List of excluded full-text articles with reasons for exclusion

| **#** | **Authors** | **Year** | **Title** | **Reason for exclusion** |
| --- | --- | --- | --- | --- |
| 1 | J. Secnik et al. | 2024 | Glucose-Lowering Medications and Post-Dementia Survival in Patients with Diabetes and Dementia | Unrelated outcome |
| 2 | J. Secnik et al. | 2020 | Dementia Diagnosis Is Associated with Changes in Antidiabetic Drug Prescription: An Open-Cohort Study of ~130,000 Swedish Subjects over 14 Years | Unrelated outcome |
| 3 | Y. Oe et al. | 2024 | Efficacy and safety of oral semaglutide in older patients with type 2 diabetes: a retrospective observational study (the OTARU-SEMA study) | Unrelated outcome |
| 4 | J. Jiang et al. | 2021 | Comparison of dapaglifozin and liraglutide in patients with poorly controlled type 2 diabetes mellitus: A 24-week, open, double-centered, head to head trial | Unrelated outcome |
| 5 | K. A. Han et al. | 2024 | Dual add-on therapy of gemigliptin and dapagliflozin in patients with type 2 diabetes inadequately controlled with metformin alone: The SOLUTION 2 study | Unrelated outcome |
| 6 | C. Gómez-Martínez et al. | 2024 | Glycated hemoglobin, type 2 diabetes, and poor diabetes control are positively associated with impulsivity changes in aged individuals with overweight or obesity and metabolic syndrome | Unrelated outcome |
| 7 | F. Formiga et al. | 2023 | Diabetes and factors associated with cognitive and functional decline. The screening for CKD among older people across Europe (SCOPE) study | Unrelated outcome |
| 8 | H. U. Euctr et al. | 2022 | A research study to see how well CagriSema helps people losing weight in people who have a body weight above the healthy range and type 2 diabetes | Unrelated outcome |
| 9 | H. U. Euctr et al. | 2016 | Empagliflozin and its effect on heart failure in type 2 diabetes | Unrelated outcome |
| 10 | D. Edmonston et al. | 2024 | Kidney and Cardiovascular Effectiveness of SGLT2 Inhibitors vs GLP-1 Receptor Agonists in Type 2 Diabetes | Unrelated outcome |
| 11 | E. Al-Ozairi et al. | 2024 | Obesity Treatments to Improve Type 1 Diabetes (OTID): a randomized controlled trial of the combination of glucagon-like peptide 1 analogues and sodium-glucose cotransporter 2 inhibitors—protocol for Obesity Treatments to Improve Type 1 Diabetes (the OTID trial) | Unrelated outcome |
| 12 | Yan Bi et al. | 2022 | Evaluating the Effects of Liraglutide, Empagliflozin and Linagliptin on Mild Cognitive Impairment Remission in Patients With Type 2 Diabetes: a Multi-center, Randomized, Parallel Controlled Clinical Trial With an Extension Phase | Incomplete/insufficient data |
| 13 | Zhiming Zhu et al. | 2022 | Effects of SGLT2i on the Cognitive Function in T2DM Patient (ESCDP) | Incomplete/insufficient data |
| 14 | ChiCtr et al. | 2022 | Clinical efficacy and safety study of SGLT-2 inhibitor dagliflozin to improve cognitive dysfunction in combination with type 2 diabetes | Incomplete/insufficient data |
| 15 | H. Cheng et al. | 2022 | Enhancement of Impaired Olfactory Neural Activation and Cognitive Capacity by Liraglutide, but Not Dapagliflozin or Acarbose, in Patients With Type 2 Diabetes: A 16-Week Randomized Parallel Comparative Study | Incomplete/insufficient data |
| 16 | H. Akimoto et al. | 2020 | Antidiabetic Drugs for the Risk of Alzheimer Disease in Patients With Type 2 DM Using FAERS | Incomplete/insufficient data |
| 17 | S. T. Osman et al. | 2025 | Positive impact of DPP-4 or SGLT2 inhibitors on mild cognitive impairment in type 2 diabetes patients on metformin therapy: A metabolomic mechanistic insight | Intervention/control: non SGLT2is |
| 18 | C. H. Nørgaard et al. | 2022 | Treatment with glucagon-like peptide-1 receptor agonists and incidence of dementia: Data from pooled double-blind randomized controlled trials and nationwide disease and prescription registers | Intervention/control: non SGLT2is |
| 19 | Y.Y Chen et al. | 2024 | The impact of sodium-glucose co-transporter-2 inhibitors on dementia and cardiovascular events in diabetic patients with atrial fibrillation | Intervention/control: non GLP-1RA/DPP4is |

**Supplementary Table S4**. Detailed study protocol and baseline population characteristics of included studies

| study | Subgroup analysis | Analysis model | Weighting method | Concominant drugs |
| --- | --- | --- | --- | --- |
| Wu et al. (2023) | ∙ Age: 66-75 vs ≥75  ∙ Sex  ∙ Comparison between individual SGLT2 inhibitors  ∙ Follow-up duration: 2.8yr | ITT and AT | IPTW based on Propensity scores | insulin acarbose metformin repaglinide rosiglitazone semaglutide pioglitazone gliclazide glyburide |
| Shin et al. (2024) | ∙ Age: 50-64 vs ≥60  ∙ Sex  ∙ Follow-up duration: 1.8yr  ∙ Baseline CV risk | ITT and AT | Propensity scrore match(PSM)  1:1 | insulin biguanide GLP1RA sulfonylurea glinides thiazolidinedione a-glucosidase |
| Perna et al. (2018) | NA | AT | NA | metformin |
| Pai et al. (2024) | ∙ Age: 50-66 vs ≥65  ∙ Sex  ∙ Follow-up duration: 690days(SGLT2i), 1,253days(DPP-4i) | ITT | Propensity score match(PSM)  1:1 | metformin |
| Mui et al. (2021) | ∙ Follow-up duration: 472days  ∙ CVD history | ITT and AT | Propensity score match(PSM)  1:2 | Beta blockers Diuretics Anticoagulants Antiplatelets Antihypertensive drugs Lipid lowering drugs Statins and filbrates Non-steroidal anti-inflammatory drugs Other antidiabetic drugs |
| Hong et al. (2024) (vs DPP-4i) | ∙ Age: 40-65 vs ≥65  ∙ Sex  ∙ Comparison between individual SGLT2 inhibitors  ∙ Follow-up duration: 4.8 yr  ∙ CVD history | ITT | PS fine stratification | beta-blockers ACEi/ARB Calcium channel blockers Diuretics Lipid lowering drugs Nitrates Anticoagulant Antiplatelets Corticosteroids Antibiotics NSAIDs |
| Hong et al. (2024) (vs GLP-1RA) | ∙ Age: 60-75 vs ≥75  ∙ Sex  ∙ Follow-up duration: 2yr  ∙ CVD history | ITT | Propensity score match(PSM) | beta-blockers ACEi/ARB CCB lipid lowering drugs nitrates anticoagulants antiplatelets corticosteroids antibiotics NSAIDs antidepressants antipsychotics anxiolytics hypnotics and sedatives psychostimulants |
| De Giorgi et al. (2024) | ∙ Age: 18-65 vs ≥65  ∙ longer follow-up of 2yrs | AT | Propensity score match(PSM) 1:1 | insulin metformin beta-blockers CCBs RAAS inhibitors statins corticosrteroids thyroid agents anti-infectives anti-neoplatics anti-imflammatories opioids antimigraine antiepileptics antipsychotics  hypnotics/sedatives SSRIs other antidepressants |
| Abdullah et al. (2025) | ∙ Age: 40-65 vs ≥65  ∙ Sex  ∙ Comparison between individual SGLT2 inhibitors  ∙ CVD history | AT | PS fine stratification | metformin Sulfonlyurease Meglitinides Thiazolidinediones Alpha-glucosidase inhibitors GLP1 receptor agonists Insulin Lipid lowering therapy Anticoagulation therapy Antihypertensive therapy |

**Abbreviations**: AD, Alzheimer’s disease; AGI, alpha-glucosidase inhibitor; AT, as-treated; CCB, calcium channel blocker; CVD, cardiovascular disease; GLP-1RA, glucagon-like peptide-1 receptor agonist; ITT, intention-to-treat; IPTW, inverse probability of treatment weighting; MCI, mild cognitive impairment; NSAIDs, nonsteroidal anti-inflammatory drugs; PSM, propensity score matching; RAAS, renin–angiotensin–aldosterone system; RASI, renin–angiotensin system inhibitors; SGLT2i, sodium-glucose cotransporter 2 inhibitors; SSRI, selective serotonin reuptake inhibitor; TZD, thiazolidinediones; VD, vascular dementia.

**Supplementary Table S5.** Detailed information on the risk of bias assessment according to the Risk of Bias 2 (ROB2) criteria

| **Study**  **(author, year)** | **Risk of bias domain** | **Assessment of risk of bias** | **The reason for judgment** |
| --- | --- | --- | --- |
| Perna  2018 | Randomization process | Some concerns | The absence of allocation concealment information results in some concerns regarding the risk of bias. |
|  | Deviations from the intended intervention | Low | Since the study adhered to intention-to-treat principles and no deviations from the intervention were reported, the study shows a low risk of bias. |
|  | Missing outcome data | Low | The study retained outcome data for most participants, with transparent reporting of dropouts. |
|  | Measurement of  the outcome | Some concerns | The measurement methods were appropriate and standardized across groups, reducing potential bias. However, the lack of blinding for outcome assessors introduces some concerns. |
|  | Selection of the reported result | Low | The study outcomes were clearly defined, and there is no evidence of selective reporting from multiple eligible measurements or analyses. |
|  | Overall | Some concerns | The study has a low risk of bias in most domains, but some concerns arise due to the lack of information on allocation concealment and blinding for outcome assessors. |

**Supplementary Table S6.** Detailed information regarding the risk of bias assessment according to the Risk of Bias in Non-randomized Studies-of Interventions (ROBINS-I)

| **Study**  **(author, year)** | **Risk of bias domain** | **Assessment of risk of bias** | **Reasons for judgment** |
| --- | --- | --- | --- |
| Wu et al.  2023 | Bias due to confounding | Moderate  (Low) | The study has a Low risk of bias, with some concerns regarding uncontrolled confounding due to missing confounding data. |
|  | Bias in the selection of participants in the study | Moderate  (Low) | The intervention status was accurately classified for nearly all participants, with minimal risk of misclassification bias, leading to a low risk of bias. |
|  | Bias in the classification of interventions | Low | The study consistently addressed and mitigated risks of selection bias through robust design and statistical adjustments. |
|  | Bias due to deviations from intended interventions | Low | The study adhered to real-world observational practices and appropriate analytical methods were used to address potential biases due to deviations. |
|  | Bias due to missing data | Low | The study has a low risk of bias due to missing data, with minimal missingness in intervention status, outcomes, and confounders. |
|  | Bias in the measurement of outcomes | Low | The study relied on standardized data with consistent outcome measurement methods across intervention groups. |
|  | Bias in the selection of the reported result | Low | The study's results were reported transparently, with no evidence of selective reporting in outcomes, analyses, or subgroups. |
|  | Overall | Moderate | There is the possibility of uncontrolled confounding that has not been controlled for, but otherwise little or no concern about bias in the result |
| Shin et al.  2024 | Bias due to confounding | Moderate  (Low) | Baseline confounding was well controlled through PSM, but time-varying confounding was not explicitly adjusted using advanced statistical methods. |
|  | Bias in the selection of participants in the study | Low | The intervention status was accurately classified for nearly all participants, with minimal risk of misclassification bias, leading to a low risk of bias. |
|  | Bias in the classification of interventions | Low | The study consistently addressed and mitigated risks of selection bias through robust design and statistical adjustments. |
|  | Bias due to deviations from intended interventions | Low | The study primarily used an As-Treated analysis with censoring upon treatment changes, risking informative censoring. A secondary ITT analysis confirmed robustness, but time-varying adherence was not explicitly modeled. |
|  | Bias due to missing data | Low | The study has a low risk of bias due to missing data, with minimal missingness in intervention status, outcomes, and confounders. |
|  | Bias in the measurement of outcomes | Low | The study relied on standardized data with consistent outcome measurement methods across intervention groups. |
|  | Bias in the selection of the reported result | Low | The study's results were reported transparently, with no evidence of selective reporting in outcomes, analyses, or subgroups. |
|  | Overall | Moderate | There is the possibility of uncontrolled confounding that has not been controlled for, but otherwise little or no concern about bias in the result |
| Pai et al.  2024 | Bias due to confounding | Moderate | The study controlled key confounders using PSM and adjusted for multiple baseline factors. While some residual confounding exists due to unmeasured variables, its impact is likely minimal. No major concerns from post-intervention variables or unmeasured confounding were identified. |
|  | Bias in the selection of participants in the study | Moderate  (Low) | The intervention status was accurately classified for nearly all participants, with minimal risk of misclassification bias, leading to a low risk of bias. |
|  | Bias in the classification of interventions | Low | The study consistently addressed and mitigated risks of selection bias through robust design and statistical adjustments. |
|  | Bias due to deviations from intended interventions | Moderate  (Low) | The study adhered to real-world observational practices and appropriate analytical methods were used to address potential biases due to deviations. |
|  | Bias due to missing data | Low | The study has a low risk of bias due to missing data, with minimal missingness in intervention status, outcomes, and confounders. |
|  | Bias in the measurement of outcomes | Low | The study relied on standardized data with consistent outcome measurement methods across intervention groups. |
|  | Bias in the selection of the reported result | Low | The study's results were reported transparently, with no evidence of selective reporting in outcomes, analyses, or subgroups. |
|  | Overall | Moderate | There is the possibility of uncontrolled confounding that has not been controlled for, but otherwise little or no concern about bias in the result |
| Mui et al.  2021 | Bias due to confounding | Moderate | The study controlled key confounders using PSM and adjusted for multiple baseline factors. While some residual confounding exists due to unmeasured variables, its impact is likely minimal. No major concerns from post-intervention variables or unmeasured confounding were identified. |
|  | Bias in the selection of participants in the study | Moderate  (Low) | The intervention status was accurately classified for nearly all participants, with minimal risk of misclassification bias, leading to a low risk of bias. |
|  | Bias in the classification of interventions | Low | The study consistently addressed and mitigated risks of selection bias through robust design and statistical adjustments. |
|  | Bias due to deviations from intended interventions | Moderate  (Low) | The study adhered to real-world observational practices and appropriate analytical methods were used to address potential biases due to deviations. |
|  | Bias due to missing data | Low | The study has a low risk of bias due to missing data, with minimal missingness in intervention status, outcomes, and confounders. |
|  | Bias in the measurement of outcomes | Low | The study relied on standardized data with consistent outcome measurement methods across intervention groups. |
|  | Bias in the selection of the reported result | Low | The study's results were reported transparently, with no evidence of selective reporting in outcomes, analyses, or subgroups. |
|  | Overall | Moderate | There is the possibility of uncontrolled confounding that has not been controlled for, but otherwise little or no concern about bias in the result |
| Hong et al.  2024  (DPP-4i) | Bias due to confounding | Moderate | The study has a Moderate risk of bias, with some concerns regarding uncontrolled confounding due to missing confounding data. |
|  | Bias in the selection of participants in the study | Moderate  (Low) | The intervention status was accurately classified for nearly all participants, with minimal risk of misclassification bias, leading to a low risk of bias. |
|  | Bias in the classification of interventions | Low | The study consistently addressed and mitigated risks of selection bias through robust design and statistical adjustments. |
|  | Bias due to deviations from intended interventions | Low | The study adhered to real-world observational practices and appropriate analytical methods were used to address potential biases due to deviations. |
|  | Bias due to missing data | Low | The study has a low risk of bias due to missing data, with minimal missingness in intervention status, outcomes, and confounders. |
|  | Bias in the measurement of outcomes | Low | The study relied on standardized data with consistent outcome measurement methods across intervention groups. |
|  | Bias in the selection of the reported result | Low | The study's results were reported transparently, with no evidence of selective reporting in outcomes, analyses, or subgroups. |
|  | Overall | Moderate | There is the possibility of uncontrolled confounding that has not been controlled for but otherwise little or no concern about bias in the result |
| Hong et al.  2024  (GLP-1RA) | Bias due to confounding | Moderate  (Low) | The study has a Low risk of bias, with some concerns regarding uncontrolled confounding due to missing confounding data. |
|  | Bias in the selection of participants in the study | Moderate  (Low) | The intervention status was accurately classified for nearly all participants, with minimal risk of misclassification bias, leading to a low risk of bias. |
|  | Bias in the classification of interventions | Low | The study consistently addressed and mitigated risks of selection bias through robust design and statistical adjustments. |
|  | Bias due to deviations from intended interventions | Low | The study adhered to real-world observational practices and appropriate analytical methods were used to address potential biases due to deviations. |
|  | Bias due to missing data | Low | The study has a low risk of bias due to missing data, with minimal missingness in intervention status, outcomes, and confounders. |
|  | Bias in the measurement of outcomes | Low | The study relied on standardized data with consistent outcome measurement methods across intervention groups. |
|  | Bias in the selection of the reported result | Low | The study's results were reported transparently, with no evidence of selective reporting in outcomes, analyses, or subgroups. |
|  | Overall | Moderate | There is the possibility of uncontrolled confounding that has not been controlled for but otherwise little or no concern about bias in the result |
| De Giorgi et al.  2024 | Bias due to confounding | Moderate | The study has a Moderate risk of bias, with some concerns regarding uncontrolled confounding due to missing confounding data. |
|  | Bias in the selection of participants in the study | Moderate  (Low) | The intervention status was accurately classified for nearly all participants, with minimal risk of misclassification bias, leading to a low risk of bias. |
|  | Bias in the classification of interventions | Low | The study consistently addressed and mitigated risks of selection bias through robust design and statistical adjustments. |
|  | Bias due to deviations from intended interventions | Moderate  (Low) | The study adhered to real-world observational practices and appropriate analytical methods were used to address potential biases due to deviations. |
|  | Bias due to missing data | Low | The study has a low risk of bias due to missing data, with minimal missingness in intervention status, outcomes, and confounders. |
|  | Bias in the measurement of outcomes | Low | The study relied on standardized data with consistent outcome measurement methods across intervention groups. |
|  | Bias in the selection of the reported result | Low | The study's results were reported transparently, with no evidence of selective reporting in outcomes, analyses, or subgroups. |
|  | Overall | Moderate | There is the possibility of uncontrolled confounding that has not been controlled for but otherwise little or no concern about bias in the result |
| Abdullah et al.  2024 | Bias due to confounding | Critical | Key confounders such as baseline cognitive function, education level, and family history of dementia were not measured or adjusted for, leading to a critical risk of bias due to confounding. |
|  | Bias in the selection of participants in the study | Moderate  (Low) | The intervention status was accurately classified for nearly all participants, with minimal risk of misclassification bias, leading to a low risk of bias. |
|  | Bias in the classification of interventions | Low | The study consistently addressed and mitigated risks of selection bias through robust design and statistical adjustments. |
|  | Bias due to deviations from intended interventions | Low | The study handled deviations through an As-Treated approach, censoring, and sensitivity analyses. |
|  | Bias due to missing data | Low | The study has a low risk of bias due to missing data, with minimal missingness in intervention status, outcomes, and confounders. |
|  | Bias in the measurement of outcomes | Low | The study relied on standardized data with consistent outcome measurement methods across intervention groups. |
|  | Bias in the selection of the reported result | Low | The study's results were reported transparently, with no evidence of selective reporting in outcomes, analyses, or subgroups. |
|  | Overall | Critical | Due to serious risk of residual confounding from unmeasured variables such as cognitive status and socioeconomic factors, the study was judged to have a critical risk of bias overall. |

# Supplementary Figures

ITT

(a) Incretin mimetics

AT

(b) DPP-4i

ITT

AT

(c)GLP-1RA

ITT

**Supplementary Figure S1.** Subgroup analysis of dementia risk: Intention-to-Treat (ITT) vs. As-Treated (AT) approaches


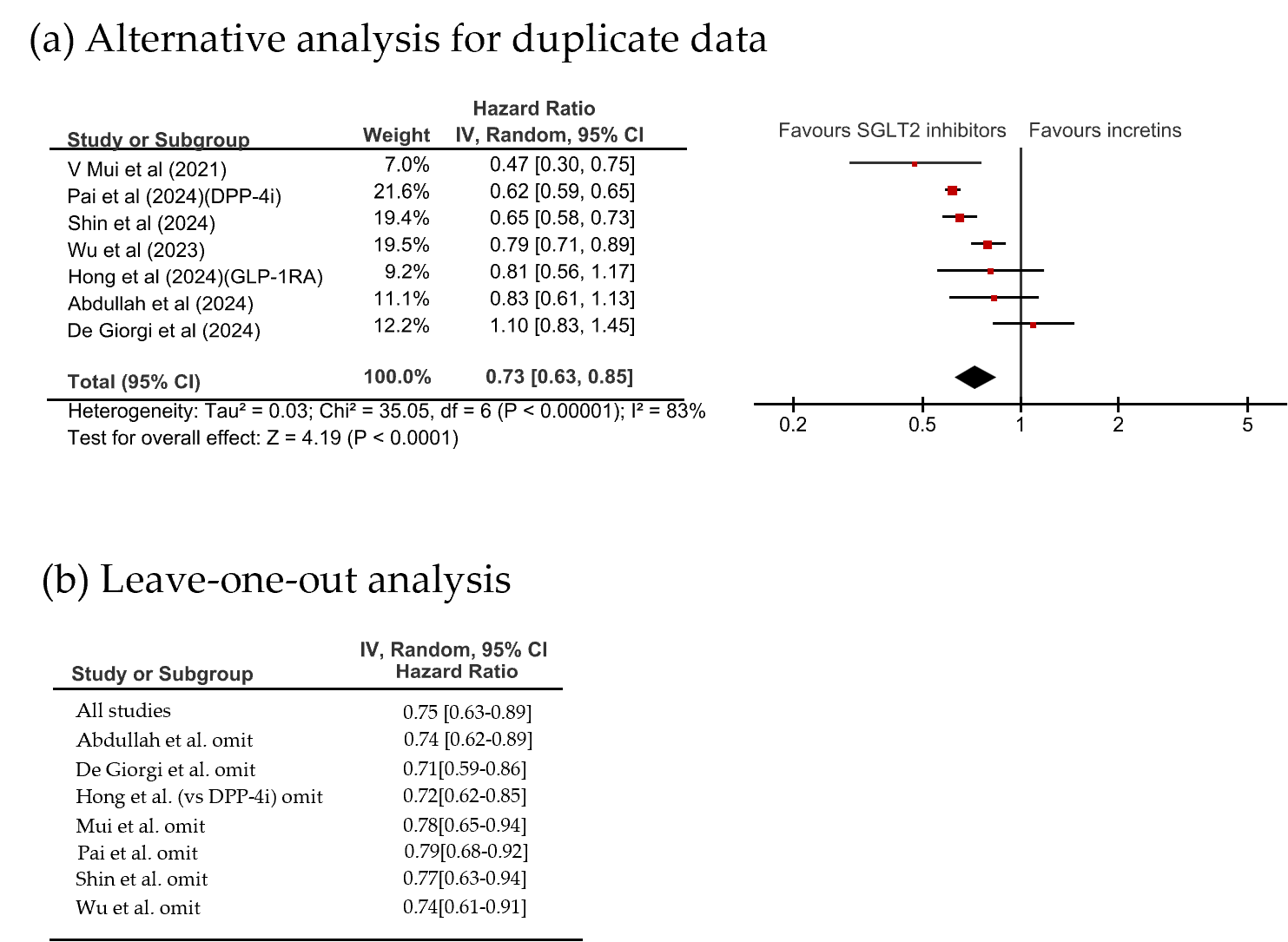


**Supplementary Figure S2**. Sensitivity analysis of new-onset dementia outcome

(1) Forest plot of new- onset of dementia with SGLT2i compared to: (a) incretin mimetics, (b) DPP-4i

1. Incretin mimetics – critical out
2. DPP-4i – critical out

(2) Forest plot of vascular dementia, Alzheimer’s disease incidence with SGLT2i

1. Vascular dementia compared to incretin mimetics - critical out
2. Alzheimer’s disease compared to incretin mimetics – critical out
3. Alzheimer’s disease compared to DPP-4i – critical out

**Supplementary Figure S3.** Sensitivity analysis excluding studies with high risk of bias


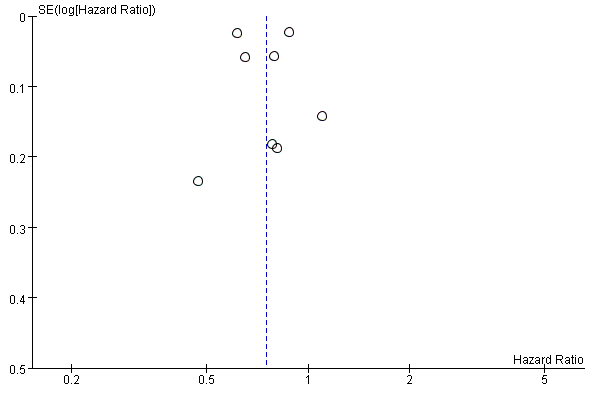


**Supplementary Figure S4.** Funnel plot of included studies evaluating SGLT2is versus incretin-based therapies for incident dementia
